# Thermal Strain Analysis of Optic Fiber Sensors

**DOI:** 10.3390/s130201846

**Published:** 2013-01-31

**Authors:** Shiuh-Chuan Her, Chih-Ying Huang

**Affiliations:** Department of Mechanical Engineering, Yuan Ze University, Chung-Li 320, Taiwan; E-Mail: s907219@mail.yzu.edu.tw

**Keywords:** optic fiber sensor, thermal strain, thermal expansion coefficient

## Abstract

An optical fiber sensor surface bonded onto a host structure and subjected to a temperature change is analytically studied in this work. The analysis is developed in order to assess the thermal behavior of an optical fiber sensor designed for measuring the strain in the host structure. For a surface bonded optical fiber sensor, the measuring sensitivity is strongly dependent on the bonding characteristics which include the protective coating, adhesive layer and the bonding length. Thermal stresses can be generated due to a mismatch of thermal expansion coefficients between the optical fiber and host structure. The optical fiber thermal strain induced by the host structure is transferred via the adhesive layer and protective coating. In this investigation, an analytical expression of the thermal strain and stress in the optical fiber is presented. The theoretical predictions are validated using the finite element method. Numerical results show that the thermal strain and stress are linearly dependent on the difference in thermal expansion coefficients between the optical fiber and host structure and independent of the thermal expansion coefficients of the adhesive and coating.

## 1. Introduction

The use of optical fiber sensors in structural health monitoring has increased rapidly. These sensors can be used to monitor both strain and damage development [1–3]. This ability is essentially due to their capability of performing direct strain measurements inside host structures at desired locations, thus providing valuable information on the local strain state within complex structures. The primary benefits of optical fiber sensors over conventional electrical sensors are their light weight, high temperature endurance, dielectric nature, immunity to electromagnetic interference and excellent multiplexing capabilities. Optical fiber sensors based on the fiber Bragg grating (FBG) have been demonstrated successively in monitoring structures. Mulle *et al.* [4] used FBG sensors to measure the residual strain in carbon-epoxy composite laminates. Zhang *et al.* [5] proposed an improved FBG sensor for the simultaneous measurements of force and temperature. Interferometric type fiber optical sensors have the advantages of high sensitivity and potentially high spatial resolution, therefore, they can be used for health monitoring in composite structures [6,7]. As a sensor, it is expected that the strains between the optical fiber and host structure are the same. However, due to the existence of the adhesive layer and protective coating, part of the energy would convert into shear deformation. Thus, the strain of the optical fiber would be different from that in the host structure. Lau *et al.* [8] developed a simple model to calculate the percentage of strain applied to the host structure actually transferred to the embedded fiber optical sensor. For optical fiber strain sensors to be accurate and practical, it is necessary to separate the mechanical strain from the thermal effect. Several techniques have been proposed for the mechanical strain and temperature discrimination, such as the reference-grating technique [9], the dual-wavelength technique [10] and the hybrid-grating technique [11]. To separate the mechanical strain from the temperature effect, it is necessary to determine the thermal strain of the optical fiber sensor when the host structure is subjected to a temperature change. Lu *et al.* [12] investigated theoretically and experimentally the distribution of thermal residual strain in optical fibers based on Brillouin optical time-domain reflectometry system. Lo and Chuang [13] measured the thermal expansion coefficient using a surface-mounted FBG sensor. Mueller *et al.* [14] proposed a high-precision thermal strain measurement model using surface bonded FBG sensors. Kim *et al.* [15] used FBG sensors to measure the thermal deformation of space structures by installing the test specimen in a vacuum chamber to simulate space environment. Yablon [16] revealed the influences of frozened-in stresses and strains on the optical and mechanical performance of optical fibers. Kim *et al.* [17] proposed a new FBG model to investigate the effect of transverse strain on the measurement of thermal strain in composite materials. Yoon *et al.* [18] performed experimental test to valid this model by measuring thermal expansion of anisotropic composite specimens and an isotropic invar specimen. In this investigation, the optical fiber sensor is surface bonded onto the host structure. An analytical expression of the thermal strain in the optical fiber induced by the host structure is presented. The theoretical prediction of the thermal strain in the surface bonded optical fiber sensor is validated using the finite element method.

## 2. Thermal Analysis

In this investigation, the thermal strain of the optical fiber induced by the temperature change of the host structure is derived based on the following assumptions:

1. All interfaces are perfectly bonded *i.e.*, displacement continuity along the interface.
2. The thermal strain of the host structure due to the temperature change is transferred to the optical fiber via the adhesive layer and protective coating. This assumption is reasonable since the Young's moduli of the coating and adhesive are significantly smaller than that for the host structure and optical fiber.
3. The temperature change in the host structure is uniform.
4. All the material properties remain constant during the temperature change.

**Figure 1.**
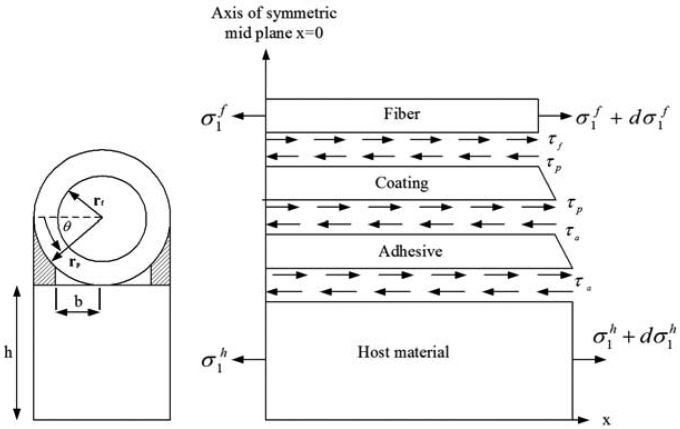
Analytical model of surface bonded optical fiber.

The analytical model is shown in Figure 1 with a cylindrical optical fiber and coating on the top and the host material under the bottom with adhesive in between. The host material is subjected to an uniform temperature change of Δ*T*. Thermal stresses can be generated due to the mismatch in thermal expansion coefficients between the optical fiber and host structure. The thermal strain of the optical fiber induced by the host structure due to the temperature change is transferred via the adhesive layer and protective coating.

The equilibrium equation for coating is:

(1) $$r_{p}\cdot \int_{0}^{2\pi }\tau_{p}(r_{p},\theta ,x)d\theta \cdot dx-r_{f}\cdot \int_{0}^{2\pi }\tau_{p}(r_{f},\theta ,x)d\theta \cdot dx=0$$

where *r_p_* and *r_j_* are the radii of the coating and optical fiber, respectively; *τ_p_* represents the shear stress in the coating which is inverse to the radius and can be expressed as:

(2) $$\tau_{p}(r,\theta ,x)=\frac{r_{p}}{r}\tau_{p}(r_{p},\theta ,x)$$

The shear strain in the coating is:

(3) $$\gamma_{p}=\frac{\partial u_{\sigma }^{p}(r,\theta ,x)}{\partial r}=\frac{\tau_{p}(r,\theta ,x)}{G_{p}}$$

where $u_{\sigma }^{p}$ is the mechanical displacement of the coating induced by the thermal stress.

Substituting Equation (3) into Equation (2), yields:

(4) $$\frac{\partial u_{\sigma }^{p}(r,\theta ,x)}{\partial r}=\frac{1}{G_{p}}\cdot \frac{r_{p}}{r}\tau_{p}(r_{p},\theta ,x)$$

where *G_p_* represent the shear modulus of the coating. Integration with respective to radius gives:

(5) $$u_{\sigma }^{p}(r,\theta ,x)=\frac{r_{p}}{G_{p}}\cdot \ln(r)\cdot \tau_{p}(r_{p},\theta ,x)+C_{1}$$

Enforcing the displacement continuity at the interface between the coating and optical fiber, yields:

$$u_{\sigma }^{p}(r_{f},\theta ,x)+\int \alpha_{p}\Delta \text{Tdx}=u_{\sigma }^{f}(r_{f},\theta ,x)+\int \alpha_{f}\Delta \text{Tdx}$$

where $u_{\sigma }^{f}$ represents the mechanical displacement of the optical fiber induced by the thermal stress; *α_p_* and *α_f_* denote the thermal expansion coefficients of the coating and optical fiber, respectively.

The mechanical displacement of the coating Equation (5) can be rewritten as:

(6) $$u_{\sigma }^{p}(r,\theta ,x)=\frac{r_{p}}{G_{p}}\cdot \ln\left(\frac{r}{r_{f}}\right)\cdot \tau_{p}(r_{p},\theta ,x)+u_{\sigma }^{f}(r_{f},\theta ,x)+\int \alpha_{f}\Delta \text{Tdx}-\int \alpha_{p}\Delta \text{Tdx}$$

where $u_{\sigma }^{f}(r_{f},\theta ,x)$ is the mechanical displacement of the optical fiber induced by the thermal stress. The displacement continuity at the interface between the coating and adhesive can be written as:

(7) $$u_{\sigma }^{p}(r_{p},\theta ,x)+\int \alpha_{p}\Delta \text{Tdx}=u_{\sigma }^{a}(r_{p},\theta ,x)+\int \alpha_{a}\Delta \text{Tdx}$$

where $u_{\sigma }^{a}$ represents the mechanical displacement of the adhesive induced by the thermal stress; *α_α_* denote the thermal expansion coefficients of the adhesive.

Substituting Equation (6) into Equation (7) yields:

(8) $$u_{\sigma }^{a}(r_{p},\theta ,x)=\frac{r_{p}}{G_{p}}\cdot \ln\left(\frac{r_{p}}{r_{f}}\right)\cdot \tau_{p}(r_{p},\theta ,x)+u_{\sigma }^{f}(r_{f},\theta ,x)+\int \alpha_{f}\Delta \text{Tdx}-\int \alpha_{a}\Delta \text{Tdx}$$

The thickness of adhesive is angular dependent as shown in Figure 1:

(9) $$t(\theta )=r_{p}-r_{p}\text{sin}\;\theta$$

The shear strain and stress in the adhesive are:

(10a) $$\gamma_{a}=\frac{u_{\sigma }^{h}+\int \alpha_{h}\Delta \text{Tdx}-u_{\sigma }^{a}(r_{p},\theta ,x)-\int \alpha_{a}\Delta \text{Tdx}}{r_{p}-r_{p}\sin\theta }$$

(10b) $$\tau_{a}=\frac{u_{\sigma }^{h}+\int \alpha_{h}\Delta \text{Tdx}-u_{\sigma }^{a}(r_{p},\theta ,x)-\int \alpha_{a}\Delta \text{Tdx}}{r_{p}-r_{p}\sin\theta }G_{a}$$

where $u_{\sigma }^{h}$ represents the mechanical displacement of the host material induced by the thermal stress; *α_h_* and Δ*T* denote the thermal expansion coefficient of the host structure and temperature change, respectively.

Substituting Equation (8) into Equation (10b) yields:

(11) $$\tau_{a}=\frac{G_{a}}{r_{p}-r_{p}\sin\theta }[u_{\sigma }^{h}-\frac{r_{p}}{G_{p}}\ln\frac{r_{p}}{r_{f}}\tau_{p}(r_{p,}\theta ,x)-u_{\sigma }^{f}(r_{f,}\theta ,x)+\int (\alpha_{h}-\alpha_{f})\Delta \text{Tdx}]$$

The continuity of shear stress at the interface between the adhesive and coating leads:

(12) $$\tau_{p}(r_{p,}\theta ,x)=\tau_{a}=\frac{G_{a}G_{p}}{G_{p}r_{p}(1-\sin\theta )+G_{a}r_{p}\ln(r_{p}/r_{p})}[u_{\sigma }^{h}-u_{\sigma }^{f}+\int (\alpha_{h}-\alpha_{f})\Delta \text{Tdx}]$$

Substituting Equation (12) into Equation (1), yields:

(13) $$\int_{0}^{2\pi }\tau_{p}(r_{p,}\theta ,x)d\theta =\frac{r_{p}}{r_{f}}\int_{0}^{\pi }\frac{1}{\left[\frac{r_{p}(1-\sin\theta )}{G_{a}}+\frac{r_{p}}{G_{p}}\ln(\frac{r_{p}}{r_{f}})\right]}\left(\left[u_{\sigma }^{h}-u_{\sigma }^{f}+\int (\alpha_{h}-\alpha_{f})\Delta \text{Tdx}\right]\right)d\theta$$

The adhesive is filled between the host material and coating with a small gap b as shown in Figure 1. Thus Equation (13) can be rewritten as:

(14) $$\int_{0}^{2\pi }\tau_{p}(r_{f,}\theta ,x)d\theta =\frac{2r_{p}}{r_{f}}\int_{0}^{\cos^{-1}(b/r_{p})}\frac{1}{\left[\frac{r_{p}(1-\sin\theta )}{G_{a}}+\frac{r_{p}}{G_{p}}\ln(\frac{r_{p}}{r_{f}})\right]}\left(\left[u_{\sigma }^{h}-u_{\sigma }^{f}+\int (\alpha_{h}-\alpha_{f})\Delta \text{Tdx}\right]\right)d\theta$$

The equilibrium equation of optical fiber in the x-axis as shown in Figure 1 is:

(15) $$\sigma_{1}^{f}\cdot \pi \cdot r_{f}^{2}=\left(\sigma_{1}^{f}+d\sigma_{1}^{f}\right)\cdot \pi \cdot r_{f}^{2}+r_{f}\cdot \left[\int_{0}^{2\pi }\tau_{p}(r_{f},\theta ,x)d\theta \right]\cdot dx$$

Now, we differentiate Equation (15) with respect to x twice and incorporate into Equation (14), which leads to:

(16) $$\frac{d^{2}\sigma_{1}^{f}}{dx^{2}}+\frac{2r_{p}}{\pi r_{f}^{2}}(\frac{\sigma_{1}^{h}}{E_{h}}-\frac{\sigma_{1}^{f}}{E_{f}}+\alpha_{h}\Delta T-\alpha_{f}\Delta T)\int_{0}^{\cos^{-1}(\frac{b}{r_{p}})}\frac{1}{\left[\frac{r_{p}(1-\sin\theta )}{G_{a}}+\frac{r_{p}}{G_{c}}\ln(\frac{r_{p}}{r_{f}})\right]}d\theta =0$$

where $\sigma_{1}^{f}$, $\sigma_{1}^{h}$ are the thermal stresses in the optical fiber and host material, respectively; *E_f_*, and *E_h_* are the Young's moduli of the optical fiber and host material, respectively.

Thermal stresses are self-equilibrium stresses supported by a body in the absence of external loads. Enforcing the self-equilibrium equation of the system, leads to the following thermal stress relationship between the optical fiber and host material:

(17) $$\sigma_{1}^{h}=\frac{-\pi r_{f}^{2}}{2\cdot h\cdot r_{p}}\sigma_{1}^{f}$$

Substituting Equation (17) into Equation (16), yields:

(18) $$\begin{array}{c} \frac{d^{2}\sigma_{1}^{f}}{dx^{2}}-{\lambda_{1}}^{2}\sigma_{1}^{f}=-\frac{2r_{p}(\alpha_{h}-\alpha_{f})\Delta T}{\pi r_{f}^{2}}\int_{0}^{\cos^{-1}(\frac{b}{r_{p}})}\frac{1}{\frac{r_{p}(1-\sin\theta )}{G_{a}}+\frac{r_{p}}{G_{p}}\ln(\frac{r_{p}}{r_{f}})}d\theta \\ \lambda_{1}=\sqrt{\left[\frac{2r_{p}}{\pi r_{f}^{2}}(\frac{\pi r_{f}^{2}}{2hr_{p}E_{h}}+\frac{1}{E_{f}})\int_{0}^{cos^{-1}(\frac{b}{r_{p}})}\frac{1}{\frac{r_{p}(1-\text{sin}\theta )}{G_{a}}+\frac{r_{p}}{G_{p}}ln(\frac{r_{p}}{r_{f}})}d\theta \right]} \end{array}$$

The solution of differential Equation (18) can be expressed as:

(19) $$\sigma_{1}^{f}=A\cosh(\lambda_{1}x)+B\sinh(\lambda_{1}x)+\frac{2r_{p}(\alpha_{h}-\alpha_{f})\Delta T}{\pi r_{f}^{2}\lambda_{1}^{2}}\int_{0}^{\cos^{-1}(\frac{b}{r_{p}})}\frac{1}{\left[\frac{r_{p}(1-\sin\theta )}{G_{a}}+\frac{r_{p}}{G_{c}}\ln(\frac{r_{p}}{r_{f}})\right]}d\theta$$

where the constants A and B can be determined by the boundary conditions at the ends of the bonded region as follows:

(20) $$\begin{array}{cc} \sigma_{1}^{f}=0\ldots \text{x}=\pm L_{f} & L_{f}:\text{half of the bonded length} \end{array}$$

By enforcing the boundary conditions Equation (20), the thermal stress of the optical fiber Equation (19) can be obtained as:

(21) $$\sigma_{1}^{f}=\frac{(\alpha_{h}-\alpha_{f})\Delta T}{\left(\frac{\pi r_{f}^{2}}{2hr_{p}E_{h}}+\frac{1}{E_{f}}\right)}[1-\frac{\cosh(\lambda_{1}x)}{\cosh(\lambda_{1}L_{f})}]$$

The thermal strain in the optical fiber is:

(22) $$\varepsilon_{1}^{f}=\frac{(\alpha_{h}-\alpha_{f})\Delta T}{E_{f}(\frac{\pi r_{f}^{2}}{2hr_{p}E_{h}}+\frac{1}{E_{f}})}[1-\frac{\cosh(\lambda_{1}x)}{\cosh(\lambda_{1}L_{f})}]+\alpha_{f}\Delta T$$

It should be noted that the thermal stress [Equation (21)] and strain [Equation (22)] are derived on the basis of uniform temperature changes in the host structure and material properties remaining constant during the temperature change. For the optical fiber sensor, it is reasonable to assume constant material properties within the temperature range of 20∼70 °C.

## 3. Finite Element Validation

**Figure 2.**
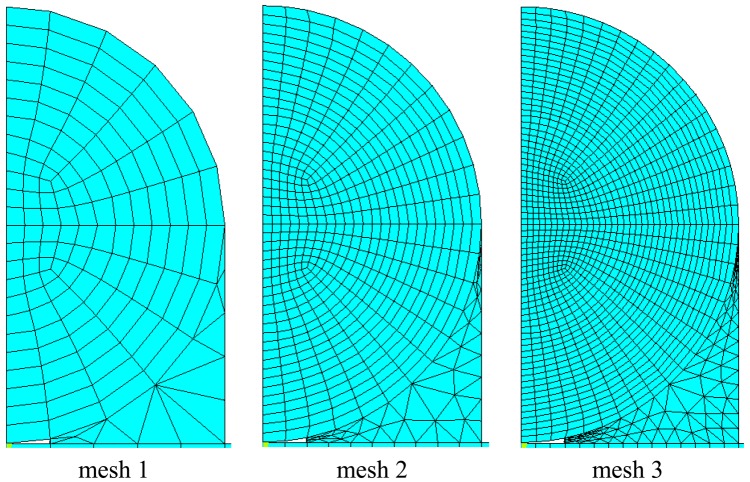
Three different finite element meshes.

The theoretical prediction of the thermal strain in the optical fiber [Equation (22)] is validated by the finite element method using the commercial software ANSYS. Eight node elements (solid 45) were used to generate meshes. Three different meshes as shown in Figure 2 are employed to conduct the convergence test. The Young's moduli for the host material, adhesive, coating and optical fiber used in the numerical study are *E_h_* = 72 GPa, *E_α_* = 2 GPa, *E_p_* = 0.0067 GPa and *E_f_* = 72 GPa, respectively; while the thermal expansion coefficients are *α_h_* = 23 με/°C, *α_α_* = 20 με/°C, *α_p_* = 76 με/°C, *α_f_* = 0.5 με/°C, respectively.

**Figure 3.**
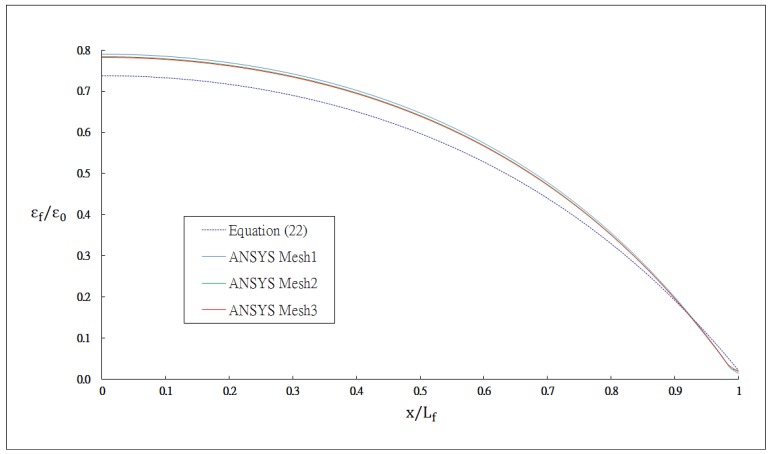
Thermal strain of the optical fiber obtained by Equation (22) and ANSYS.

**Figure 4.**
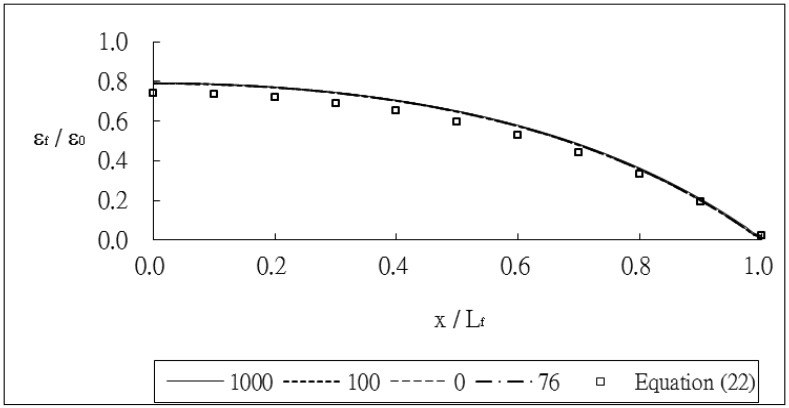
Thermal strain of the optical fiber with different thermal expansion coefficient for the coating.

**Figure 5.**
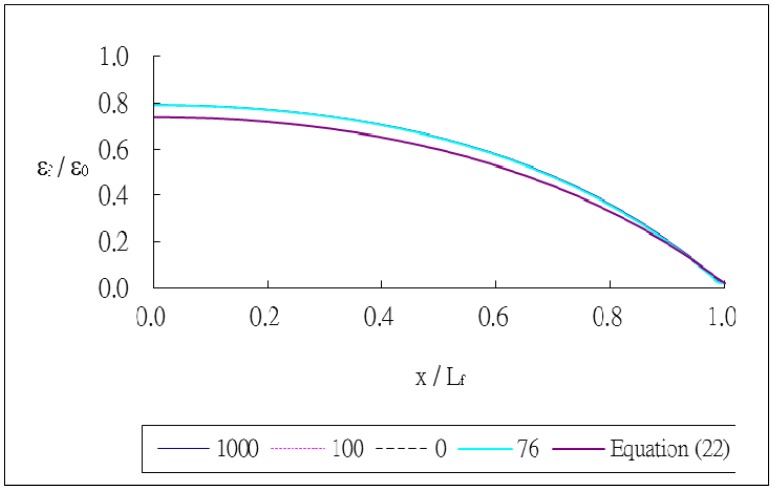
Thermal strain of the optical fiber with different thermal expansion coefficients for the adhesive.

The outer radii of the optical fiber and coating are *r_f_* = 62.5 μm and *r_p_* = 125 μm, respectively. The thickness of the host material and bonded length are *h* = 8 mm and *L_f_* = 20 mm, respectively. The index of refraction and Pockel's constants are *n_0_* = 1.45, *p_11_* = 0.12, *p_12_* = 0.27, respectively. The thermal strain of the optical fiber along the surface bonded length calculated using Equation (22) and the finite element method are plotted in Figure 3. The thermal strain *ε_f_* in the optical fiber shown in Figure 3 is normalized by the thermal strain of the host material *ε_0_* = *α_h_*Δ*T*. It shows that the theoretical predictions are in good agreement with the FEM results. A close agreement among these three different meshes demonstrates that the mesh is fine enough to converge toward the correct solution. The strain distribution of the optical fiber shows that the maximum thermal strain occurs in the middle of the surface bonded optical fiber and decreases to zero at both ends of the bonded length. The theoretical prediction of thermal strain Equation (22) is linearly dependent on the difference (*α_h_* − *α_f_*) of the thermal expansion coefficients between the host material and optical fiber and is independent of the thermal expansion coefficients of the coating and adhesive. The thermal strain of the optical fiber obtained using the finite element method with four different thermal expansion coefficients for the coating *α_p_* = 0, 76, 100 and 1,000 με/°C are presented in Figure 4. It shows that the thermal expansion coefficient of the coating does not affect the thermal strain of the optical fiber, which is in agreement with the theoretical prediction. Similar results were obtained while the thermal expansion coefficient of the adhesive was varied from *α_α_* = 0, 76, 100 and 1,000 με/°C as shown in Figure 5. In this investigation, the thermal strain is assumed to transfer from the host structure to the optical fiber via the shear deformations of the adhesive layer and protective coating. Thus, the thermal strain of the optical fiber can be dependent on the shear moduli of the adhesive layer and protective coating. Basing on the thermal analysis as described in Section 2, the thermal expansions of the adhesive and protective coating are cancelled out, leads to the optical fiber thermal strain independent of the thermal expansion coefficients of the adhesive and protective coating.

## 4. Conclusions

An analytical expression of the thermal strain of surface bonded optical fiber induced by the host structure was presented. The percentage of thermal strain in the host structure actually transferred to the optical fiber is dependent on the bonding characteristics, which include the protective coating, adhesive layer and the bonding length. Three-dimensional finite element analysis was conducted using commercial software ANSYS and compared with the theoretical prediction. Good agreement was observed between the numerical result and theoretical prediction. The parametric study showed that the thermal strain and stress are linearly dependent on the difference in thermal expansion coefficients between the optical fiber and host structure and are independent of the thermal expansion coefficients of the adhesive and coating.
